# Lemierre Syndrome Presenting With Septic Pulmonary Emboli: A Case Report and Diagnostic Challenges

**DOI:** 10.7759/cureus.109678

**Published:** 2026-05-26

**Authors:** Nikolaos I Davanellos, Despoina Paraskeva, Dimitrios Argiropoulos, Michalis Apergis, Christina Pachi, Ioannis Maragkos, Nikolaos Palyvos

**Affiliations:** 1 Department of Internal Medicine, General Hospital of Syros "Vardakeio and Proio", Ermoupoli, GRC; 2 Department of Radiology, General Hospital of Syros "Vardakeio and Proio", Ermoupoli, GRC

**Keywords:** anaerobic infection, blood culture-negative infection, diagnostic delay, fusobacterium necrophorum, internal jugular vein thrombosis, lemierre syndrome, oropharyngeal infection, septic pulmonary emboli

## Abstract

Lemierre syndrome is a rare but potentially life-threatening complication of oropharyngeal infection characterized by septic thrombophlebitis of the internal jugular vein and metastatic septic embolization. Because initial manifestations often resemble uncomplicated pharyngitis, diagnosis may be delayed and lead to significant morbidity.

We report the case of a 34-year-old, previously healthy man who presented with persistent fever, sore throat, dyspnea, and cervical tenderness despite outpatient antibiotic treatment. Laboratory evaluation demonstrated a marked inflammatory response, while non-contrast chest computed tomography revealed multiple bilateral peripheral pulmonary nodules, several exhibiting central cavitation and findings suggestive of septic pulmonary emboli. Duplex ultrasonography confirmed left internal jugular vein thrombosis. Blood cultures remained negative. Broad-spectrum antimicrobial therapy with anerobic coverage was initiated, followed by therapeutic anticoagulation after radiological confirmation of thrombosis, and the patient was subsequently transferred to a tertiary referral center for further management.

This case highlights the importance of maintaining a high index of suspicion for Lemierre syndrome in patients with persistent systemic symptoms following pharyngitis, even in the absence of positive blood cultures. Early recognition and timely imaging evaluation, together with prompt initiation of appropriate therapy, remain essential to limit disease progression.

## Introduction

Lemierre syndrome is a rare but potentially life-threatening complication of oropharyngeal infection characterized by septic thrombophlebitis of the internal jugular vein and metastatic septic embolization, most commonly involving the lungs, and is classically associated with *Fusobacterium necrophorum* [1-3]. Although rare, it remains clinically significant, as delayed recognition may lead to severe pulmonary complications, including septic pulmonary emboli, necrotizing pneumonia, pleural effusion, respiratory failure, and metastatic infection [2-5]. Emerging evidence also suggests that corticosteroid exposure during early disease stages may be associated with worse clinical outcomes in selected patients [5].

Recent reports have also highlighted culture-negative and atypical presentations of Lemierre syndrome, including cases associated with alternative pathogens and non-classical patterns of thrombophlebitis [6,7].

The initial presentation often resembles uncomplicated pharyngitis, which may result in inappropriate reassurance or incomplete treatment [3,4]. Consequently, diagnosis relies primarily on clinical suspicion supported by imaging findings rather than microbiological confirmation alone, as blood cultures may remain negative and early symptoms are frequently nonspecific [4]. Imaging, therefore, plays a central role in establishing the diagnosis and assessing disease extent.

This report highlights the diagnostic challenges of Lemierre syndrome in contemporary clinical practice, particularly in patients with negative microbiological studies and predominant pulmonary manifestations. By presenting this case, we emphasize the importance of maintaining clinical suspicion and performing timely imaging evaluation to support early diagnosis and prompt initiation of appropriate therapy.

## Case presentation

A 34-year-old, previously healthy man presented to the emergency department with a 6-day history of sore throat, dysphagia, fever, dyspnea, and tender cervical swelling. Two days before presentation, he had been evaluated at an outpatient clinic, diagnosed with pharyngitis, and prescribed azithromycin. Despite treatment, his symptoms progressed, with persistent high-grade fever and worsening respiratory discomfort.

On arrival, his temperature was 38.6 °C, heart rate 100 beats per minute, respiratory rate 23 breaths per minute, blood pressure 105/65 mmHg, and oxygen saturation 92% on room air. Physical examination revealed bilateral tender cervical lymphadenopathy and palpable cord-like structures along the course of the internal jugular veins. Oropharyngeal examination demonstrated erythematous, edematous tonsils with bilateral exudates. Lung auscultation revealed crackles at the right lung base. The remainder of the examination was unremarkable.

Initial laboratory investigations demonstrated leukocytosis with neutrophil predominance, thrombocytopenia, markedly elevated inflammatory markers, and significantly elevated D-dimer levels (Table 1). Arterial blood gas analysis demonstrated mild hypoxemia without acid-base disturbance (Table 2).

**Table 1:** Initial laboratory findings at presentation WBC, white blood cell count; CRP, C-reactive protein; γ-GT, gamma-glutamyl transferase; LDH, lactate dehydrogenase

Parameter	Value	Normal Range
WBC (×10^3^/μL)	22.61	4.0 – 10.0
Neutrophils (%)	81.8	40 – 75
Lymphocytes (%)	6.0	20 – 45
Neutrophils absolute (×10^3^/μL)	18.49	1.5 – 7.5
Monocytes (×10^3^/μL)	2.76	0.2 – 0.8
Platelets (×10^3^/μL)	64	150 – 400
CRP (mg/L)	217	< 5
Glucose (mg/dL)	114	70 – 100
Urea (mg/dL)	47	15 – 45
Creatinine (mg/dL)	1.44	0.6 – 1.2
Potassium (mmol/L)	4.4	3.5 – 5.1
Sodium (mmol/L)	134	135 – 145
Calcium (mg/dL)	8.8	8.6 – 10.2
γ-GT (U/L)	101	< 55
LDH (U/L)	387	135 – 225
D-dimer (ng/mL)	1964	< 500

**Table 2:** Arterial blood gas analysis at presentation ABG: arterial blood gas; PaO₂: partial pressure of arterial oxygen; PaCO₂: partial pressure of arterial carbon dioxide; SaO₂: arterial oxygen saturation

Parameter	Value	Normal Range
pH	7.43	7.35 – 7.45
PaCO₂	36 mmHg	35 – 45 mmHg
PaO₂	71 mmHg	80 – 100 mmHg
HCO₃⁻	23.9 mmol/L	22 – 26 mmol/L
SaO₂	92% on room air	95 – 100 %
Na⁺	134 mmol/L	135 – 145 mmol/L
K⁺	3.4 mmol/L	3.5 – 5.0 mmol/L
Ca²⁺ (ionized)	0.90 mmol/L	1.12 – 1.32 mmol/L
Glucose	117 mg/dL	70 – 110 mg/dL
Lactate	1.1 mmol/L	0.5 – 2.0 mmol/L

Two sets of peripheral blood cultures were obtained on admission before initiation of intravenous antibiotics, and remained negative. Viral and serological testing, including screening for Epstein-Barr virus infection, was also negative.

The patient had no documented history of corticosteroid exposure or immunosuppressive therapy prior to presentation.

Non-contrast chest computed tomography was performed as part of the initial diagnostic evaluation and demonstrated multiple bilateral peripheral nodular pulmonary opacities, several exhibiting central cavitation, highly suggestive of septic pulmonary emboli (Figure 1). Duplex ultrasonography of the neck demonstrated extensive thrombosis of the left internal jugular vein extending from the clavicular region to the mandibular angle, accompanied by cervical lymphadenopathy (Figure 2).

**Figure 1:**
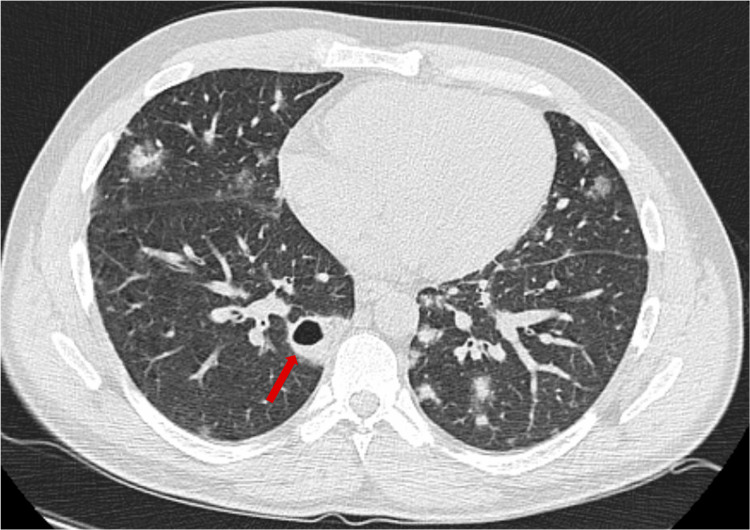
Non-contrast chest CT demonstrating bilateral septic pulmonary emboli Axial non-contrast chest CT image demonstrating multiple bilateral peripheral nodular pulmonary opacities, several exhibiting central cavitation, consistent with septic pulmonary emboli. The red arrow indicates a cavitary pulmonary lesion in the right lower lobe suggestive of septic embolic involvement.

**Figure 2:**
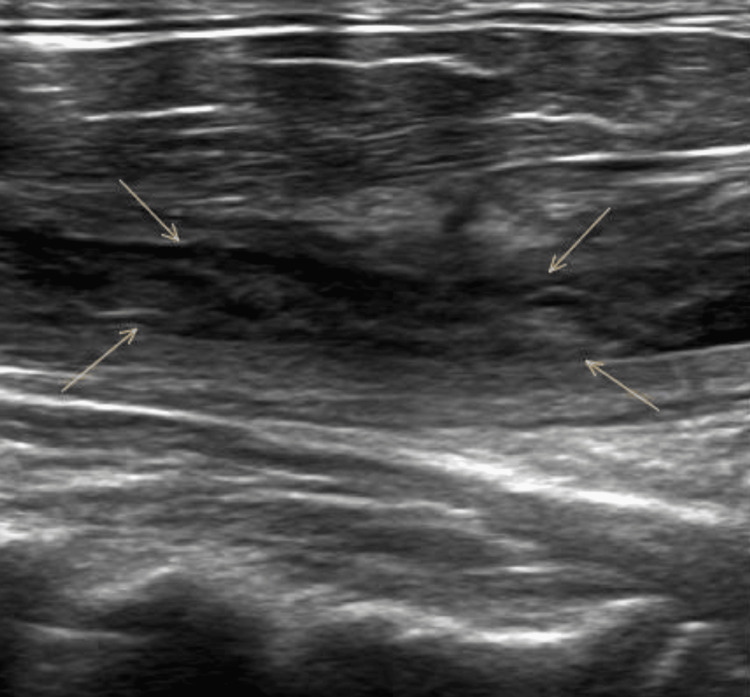
Ultrasound findings of left internal jugular vein thrombosis Longitudinal duplex ultrasonography image of the left internal jugular vein demonstrating loss of normal venous compressibility and the presence of wall-adherent echogenic intraluminal thrombus (arrows), consistent with internal jugular vein thrombosis.

Based on the combination of recent oropharyngeal infection, left internal jugular vein thrombosis, and bilateral septic pulmonary emboli, a diagnosis of Lemierre syndrome was established. The patient was admitted to the internal medicine department and received intravenous piperacillin-tazobactam together with supplemental oxygen via nasal cannula and supportive care. Therapeutic-dose subcutaneous enoxaparin (6000 IU twice daily) was initiated after confirmation of internal jugular vein thrombosis, considering the extent of thrombotic and pulmonary involvement. Following initiation of antimicrobial therapy and anticoagulation, the patient demonstrated gradual clinical improvement with stabilization of respiratory symptoms. Due to the extent of pulmonary involvement, the patient was transferred to a tertiary care center for further management.

## Discussion

Lemierre syndrome is an uncommon but potentially life-threatening complication of oropharyngeal infection, characterized by septic thrombophlebitis of the internal jugular vein and metastatic septic embolization, most frequently affecting the lungs [1-5]. Despite advances in antimicrobial therapy, delayed recognition remains common because early manifestations frequently overlap with uncomplicated pharyngitis, often resulting in delayed diagnosis and treatment [2-5].

This case illustrates several diagnostic challenges associated with Lemierre syndrome. The patient initially received outpatient treatment for presumed uncomplicated pharyngitis but subsequently developed progressive respiratory symptoms and cervical findings suggestive of systemic disease. The differential diagnosis included severe bacterial pharyngitis, deep neck space infection, infective endocarditis with septic embolization, and community-acquired pneumonia; however, the combination of recent oropharyngeal infection, internal jugular vein thrombosis, and characteristic pulmonary findings supported the diagnosis of Lemierre syndrome. Although blood cultures remained negative, likely influenced by prior antibiotic exposure, the combination of internal jugular vein thrombosis and bilateral cavitary pulmonary lesions remained highly suggestive of septic embolization and supported the final diagnosis.

Culture-negative cases and atypical presentations of Lemierre syndrome have been increasingly described, including cases associated with non-Fusobacterium pathogens, polymicrobial infection, or non-classical patterns of septic thrombophlebitis [6,7]. In the present case, although no pathogen was isolated, the clinical constellation of recent oropharyngeal infection, left internal jugular vein thrombosis, and bilateral septic pulmonary emboli remained strongly supportive of the diagnosis of Lemierre syndrome. Therefore, microbiological negativity should be interpreted in the context of prior antibiotic exposure and should not exclude the diagnosis when the clinical and radiological pattern is characteristic [4,8].

Imaging was central to establishing the diagnosis in this case. Duplex ultrasonography provided rapid confirmation of internal jugular vein thrombosis, while chest CT demonstrated characteristic bilateral peripheral nodular pulmonary lesions with cavitation, supporting septic pulmonary embolization [3,8,9]. Although contrast-enhanced imaging is often preferred for evaluating vascular complications, clinically relevant findings may still be identified using non-contrast imaging when interpreted in the appropriate clinical context. Management remains centered on prompt initiation of broad-spectrum antimicrobial therapy, whereas the role of anticoagulation continues to be individualized due to limited high-quality evidence [10,11].

This case emphasizes the importance of maintaining a high index of suspicion for Lemierre syndrome in patients presenting with persistent fever, cervical symptoms, and respiratory manifestations following recent oropharyngeal infection. Early imaging evaluation and timely initiation of empiric therapy may support timely diagnosis and optimize clinical management, particularly in secondary care settings.

## Conclusions

Lemierre syndrome remains a rare but potentially life-threatening complication of oropharyngeal infection that may initially mimic uncomplicated pharyngitis and delay diagnosis. This case highlights the importance of maintaining a high index of suspicion in patients presenting with persistent fever, cervical symptoms, and respiratory manifestations following a recent upper respiratory tract infection. Even in the absence of positive blood cultures, timely imaging evaluation and clinical-radiological correlation may facilitate early diagnosis. Prompt initiation of appropriate therapy remains essential to prevent disease progression and support favorable clinical outcomes.
